# Delivery of Phenolic Compounds, Peptides and β-Glucan to the Gastrointestinal Tract by Incorporating Dietary Fibre-Rich Mushrooms into Sorghum Biscuits

**DOI:** 10.3390/foods10081812

**Published:** 2021-08-05

**Authors:** Juncai Tu, Margaret Anne Brennan, Gang Wu, Weidong Bai, Ping Cheng, Bin Tian, Charles Stephen Brennan

**Affiliations:** 1 Department of Wine, Food and Molecular Biosciences, Lincoln University, Christchurch 7647, New Zealand; Juncai.Tu@lincolnuni.ac.nz (J.T.); Margaret.Brennan@lincoln.ac.nz (M.A.B.); Gang.Wu@lincolnuni.ac.nz (G.W.); Bin.Tian@lincoln.ac.nz (B.T.); 2 Riddet Institute, Palmerston North 4474, New Zealand; 3 College of Light Industry and Food Sciences, Zhongkai University of Agriculture and Engineering, Guangzhou 510225, China; whitebai2001@163.com (W.B.); nkpcheng@163.com (P.C.); 4 School of Science, RMIT University, GP.O. Box 2474, Melbourne, VIC 3001, Australia

**Keywords:** *Lentinula edodes*, *Auricularia auricula*, *Tremella fuciformis*, phenolic compounds, β-glucan

## Abstract

Sorghum biscuits were enriched with mushroom powders (*Lentinula edodes*, *Auricularia auricula* and *Tremella fuciformis*) at 5%, 10% and 15% substitution levels. An in vitro gastrointestinal digestion was used to evaluate the effect of this enrichment on the phenolic content and soluble peptide content as well as antioxidant activities of the gastric or intestinal supernatants (bio-accessible fractions), and the remaining portions of phenolic compounds, antioxidants and β-glucan in the undigested residue (non-digestible fraction). The phenolic content of the gastric and intestinal supernatants obtained from digested mushroom-enriched biscuits was found to be higher than that of control biscuit, and the phenolic content was positively correlated to the antioxidant activities in each fraction (*p* < 0.001). *L. edodes* and *T. fuciformis* enrichment increased the soluble protein content (small peptide) of sorghum biscuits after in vitro digestion. All mushroom enrichment increased the total phenolic content and β-glucan content of the undigested residue and they were positively correlated (*p* < 0.001). The insoluble dietary fibre of biscuits was positively correlated with β-glucan content (*p* < 0.001) of undigested residue. These findings suggested that enriching food with mushroom derived dietary fibre increases the bioavailability of the non-digestible β-glucan and phenolic compounds.

## 1. Introduction

A diet rich in biologically active ingredients (such as polyphenols and dietary fibre) can help lower the risk of chronic diseases, such as obesity, bowel inflammation and cancer and helps to regulate gut microbiota. Sorghum is rich in phenolic compounds, including phenolic acids, tannins and flavonoids, and the amount and diversity of the major polyphenols in sorghum are higher than wheat, maize and rice [1]. Previous research into the prevention of chronic disease using sorghum has concentrated on the bioactive polyphenols in relation to their effects on antioxidant capacity, oxidative stress reduction, metabolism of glucose and lipid, inflammatory activity and regulation of the gut microbiota [2]. In addition to this, sorghum is gluten-free which makes it suitable for those suffering from coeliac disease [3]. Sorghum is not a commonly-consumed cereal, but it has been reported that cereal products that claim they are a source of fibre and show potential to reduce the risk of diabetes and cardiovascular disease can gain an increase in consumer liking [4]. Therefore, sorghum has the potential to be an alternative to wheat flour traditionally used in cereal-based foods.

Sorghum has a low protein digestibility, which contributes to the hydrophobic nature of kafirins, and the way in which the proteins bind with starch granules and phenolic compounds [5,6,7,8]. Cooking increases the protein digestibility of sorghum [7], which means that biscuits could be a suitable food to deliver bioactive compounds from sorghum to the gastrointestinal tract. In common with other cereals, sorghum is deficient in lysine and is considered to have poor quality protein from a nutritional point of view [9]. Mushrooms are a good source of lysine and can be incorporated into sorghum flour to improve the protein quality [10]. They have been recognised as the only non-animal food source that can provide vitamin D, mainly in the form of D2 and D3, both of which exhibit anti-inflammation, anti-tumour and anti-cancer properties [11]. Mushrooms have a high dietary fibre content and show immunomodulatory and anti-cancer activities [12]. Mushroom β-glucan can be digested by the colonic microbiota to produce short-chain fatty acids (SCFAs) these can help to regulate blood pressure, appetite, glucose homeostasis and improve gut integrity [13]. The insoluble dietary fibre, present in mushrooms, is also fermented to produce SCFAs, however, an in vivo study using pigs showed that β-glucan had a higher fermentation rate and SCFAs production rate than other insoluble fibres [14].

It is of great interest to incorporate mushrooms into cereal products to improve the nutritional quality and functionalities of products in recent studies [15,16,17,18]. However, mushroom dietary fibre might negatively affect the bioaccessibility of the phenolic compounds and the digestion of other nutrients when they are enriched in products. They can create associations with polyphenols before and during gastrointestinal digestion [19]. Some of the phenolic compounds are available in the stomach and upper intestine to reduce the free radicals present. The remaining phenolic compounds pass into the colon and are available to be bio-transformed to metabolites via fermentation by colonic microbiota [20,21]. Research is needed to analyse the portions of the phenolic compounds and other nutrients that can reach to the upper or lower part of gastrointestinal tract and how nutrients are delivered when products are enriched by fibre-rich mushrooms.

The main aim of this study was to evaluate the effect of mushroom powder on the digestion of the developed mushroom enriched sorghum biscuits and the release of proteins and phenolic compounds in relation to the antioxidant properties. The effect of mushroom incorporation on the β-glucan content and the colonic bioavailability of phenolic compounds were evaluated.

## 2. Materials and Methods

### 2.1. Materials

Sorghum flour (Davis Trading, New Zealand) and dried shiitake (*Lentinula edodes*), black ear (*Auricularia auricula*) and silver ear (*Tremella fuciformis*) mushrooms (Jade Phoenix, Guangzhou, China) were used in this study.

### 2.2. Preparation of Biscuits

Dried mushrooms were crushed with a Coffee Grinder (Breville, Sydney, Australia) and were further processed to powder using a Laboratory Mill 3310 (PerkinElmer, Waltham, MA, USA). The dough of sorghum biscuits was prepared by mixing sorghum flour (225 g) with 65 g sugar, 64 g vegetable shortening, 2.1 g salt, 2.5 g sodium bicarbonate and 50 g distilled water with a stand mixer (Breville, Australia). The dough was rolled and cut (6 mm thickness and 57 mm diameter) before putting into an oven and bake for 15 min at 160 °C. The mushroom-enriched biscuits had 5%, 10% and 15% sorghum flour replaced with mushroom powder (As shown in Table S1).

### 2.3. In Vitro Gastrointestinal Digestion

An in vitro gastrointestinal digestion, including gastric and intestinal stage, was simulated according to the method of Wu, et al. [22]. The biscuits (2 g) were dispersed into gastric (pepsin) solution and incubated at 37 °C for 2 h. For the intestinal stage, the pH was adjusted by 2 mL of 1 mol/L NaHCO_3_ and 5 mL of 0.1 mol/L sodium maleate buffer (pH 6). After the pH adjustment, 0.1 mL of α-amyloglucosidase (3000 U/mL) was added, following by adding 5 mL of freshly prepared pancreatin-bile solution and incubated at 37 °C for 2 h. 

The samples obtained after gastric and intestinal digestion were centrifuged at 13,000× *g* for 10 min (4 °C) to separate the bio-accessible fraction (supernatant) and the undigested residue. The supernatants were used for the determination of phenolic compound content and antioxidant activity. The undigested residue was freeze-dried and ground into a powder before further evaluation. The bio-accessibility index (%) is a measure of how available the phenolic compounds for absorption [20], and it was calculated according to the equation,

$$\mathrm{BI}\;\left(\%\right)=\frac{Phenolic\;content\;of\;supernatant}{Total\;phenolic\;content\;of\;biscuit}\times 100.$$

The phenolic content in the equation was determined as described in Section 2.6.1. Total phenolic content was the sum of free and bound phenolic content.

### 2.4. BCA Assay and Protein Profile

After in vitro gastric and intestinal digestion described in Section 2.3, aliquots (0.5 mL) of digesta were taken and heated at 95 °C for 5 min. The samples were stood for 1 h at room temperature followed by centrifugation (13,000× *g*, 10 min), and the soluble protein content of the supernatant (bio-accessible fraction) was measured by Pierce™ BCA Protein Assay Kit (Thermo Fisher Scientific). The soluble protein content (SPC) was calculated according to the equation,

$$\mathrm{SPC}\;\left(\mathrm{mg}/g\;\mathrm{dw}\right)=\frac{Protein\;weight\;in\;supernatant}{Total\;dry\;weight\;of\;biscuit}\times 100.$$

An SDS-PAGE assay was carried out according to the method used by Gong, et al. [23] with NuPAGE^TM^ 4–12% Bis-Tris electrophoresis gels (Bio-Rad, Richmond, CA, USA). The marker for molecular weight (10–250 kDa) was used as a reference to the protein bands. The proteins in biscuits and digesta supernatants were extracted with the NuPAGE^TM^ LDS sample buffer and reducing agent (×1) followed by a heat treatment (100 °C for 5 min). After centrifuging, 10 μL of the maker and 20 μL of sample extracts were loaded into the gel and run at 170 V for 40 min. The gel was stained with Commassie blue G-250 for 1 h and then was destained overnight. 

### 2.5. Extraction of Phenolic Compounds

Free phenolic compounds were extracted with methanol as reported by Wang, et al. [24]. Samples (1 g) were stirred with 30 mL of 70% methanol (*v*/*v*) on a magnetic multi-stirrer overnight at ambient temperature and centrifuged for 10 min (9000× *g*, 4 °C). The supernatants were transferred to a volumetric flask (50 mL). The resulting pellets were further extracted twice with 10 mL of 70% methanol for 30 s over a vortex and the mixtures were centrifuged. The supernatants were combined and transferred quantitatively to a volumetric flask for the determination of phenolic content. 

To extract the bound phenolic compounds, the method according to Li, et al. [25] was followed. The residues obtained after methanol extractions were subjected to alkaline hydrolysis by adding 20 mL of 4 mol/L NaOH. The samples were stirred at ambient temperature for 4 h before centrifuging at 9000× *g* for 10 min. The hydrolysed samples were acidified with 5 mol/L HCl to pH 2 and then extracted with ethyl acetone four times and centrifuged. Supernatants were collected and the organic phase was evaporated under reduced pressure at 30 °C. The samples were re-dissolved in 70% methanol. All extractions were performed in triplicate and samples were kept in the dark at −20 °C prior to the determination of phenolic content.

### 2.6. Determination of Phenolic Content and Antioxidant Activity

#### 2.6.1. Phenolic Content Determination

Phenolic compound content was determined by the Folin-Ciocalteu method according to Polat, et al. [26]. The methanol extracts (free phenolic content), alkaline hydrolysed supernatants (bound phenolic content) and supernatants from the in vitro gastrointestinal digestion (bio-accessible phenolic content) were all analysed using this method. Results were expressed as milligrams of gallic acid equivalents (GAE) per gram of dry weight products.

#### 2.6.2. Antioxidant Activity

The antioxidant activity of the methanol extracts of biscuits and supernatants from the in vitro gastrointestinal digestion was determined using 2,2-diphenyl-1-picrylhydrazyl (DPPH) radical scavenging activity and ferric reducing antioxidant power (FRAP) assays according to the methods described by Wu, et al. [27]. The results were expressed as micromoles of Trolox equivalents per gram dry weight (μmol TE/g dry weight), and μmol Fe^2+^ equivalents (Fe^2+^ E)/g dry weight of samples, respectively.

### 2.7. β-Glucan Determination

The β-glucan content of the mushroom powders, biscuits and dried undigested residues were determined using the Yeast-mushroom β-glucan assay kit (Megazyme, International Ireland Ltd., Wicklow, Ireland) according to McCleary and Draga [28]. The principle of this method was to determine the total glucan and α-glucan. The β-glucan content was calculated by subtracting α-glucan content from the total glucan content.

### 2.8. Nutritional Analysis

Crude protein content was measured using the Dumas method with the conversion factor of 6.25 for biscuits. The contents of insoluble dietary fibre (IDF), soluble dietary fibre (SDF) and total dietary fibre (TDF) were evaluated using commercial Megazyme assay kits (Megazyme International Ireland Ltd., Wicklow, Ireland) based on the method of Leon Prosky, et al. [29].

### 2.9. Statistical Analysis

All samples were analysed in triplicate and recorded by mean values ± standard deviation. Significant differences between multiple mean values were analysed by the One-way ANOVA and Tukey test (*p* < 0.05) using Minitab^®^ (vision 19). Pearson’s correlation coefficients were performed using Minitab^®^ (vision 19) to assess the correlations between observed values (*p* < 0.001). Principal component analysis was conducted using Graphpad Prism 9.0 (GraphPad, CA, USA) to evaluate the effects of mushrooms substitutions on the biscuits variances.

## 3. Results and discussion

### 3.1. Phenolic Content 

The free, bound and total phenolic content of the sorghum flour, the mushroom powders and the mushroom enriched sorghum biscuits are shown in Table 1. The sorghum flour contained a total 2.98 mg GAE/g dry weight (dw) of free phenolic content (methanol extracts), and this value was significantly higher than both *A. auricula* (1.37 mg GAE/g dw) and *T. fuciformis* (1.23 mg GAE/g dw) mushrooms, but lower than *L. edodes* (7.16 mg GAE/g dw). A similar trend was observed in the total phenolic content (TPC) which was the sum of the free and bound phenolic content. The bound phenolic content of *A. auricula*, *T. fuciformis* and sorghum flour was much higher than their free phenolic content. The differences in the phenolic content of the mushrooms affected the phenolic content of the mushroom enriched sorghum biscuits.

The replacement (5–15%) of sorghum flour by mushroom powder significantly changed the phenolic content of the biscuits (*p* < 0.05). Free phenolic content of the biscuits was (*p* < 0.05) increased by the inclusion of *L. edodes* mushroom with the substitution levels, and slightly decreased by the substitution with *A. auricula* and *T. fuciformis*. The bound phenolic content of mushroom-enriched biscuits was ranged from 3.56 to 3.93 mg GAE/g dw, and the values were higher than the control biscuits (3.48 mg GAE/g dw). Biscuits enriched with *L. edodes* and *A. auricula* had an increased total phenolic content. Enrichment with *T. fuciformis* at the 5% substitution level increased the total phenolic content, but there was no significant difference at 10% and 15% levels. That means the sorghum total phenolic content was not significantly diluted by *A. auricula* and *T. fuciformis* enrichment. In bakery products, many inner physiochemical reactions related to phenolic compounds can occur upon the thermal treatment, such as the liberation of bound phenolic compounds, degradation and oxidation [30]. Previous studies reported that roasting sorghum grains at high temperatures (150 and 180 °C) led to the degradation and loss of phenolic compounds (such as gallic acid, chlorogenic acid, ellagic acid, luteolin and quercetin) [31]. The increase in bound and total phenolic content of mushroom biscuits could be due to the mushroom dietary fibres inhibiting the release of the bound phenolic compounds and the loss of free phenolic compounds during baking (160 °C). The sorghum biscuit phenolic compounds may have become attached to the mushroom dietary fibres during the biscuit making process (mixing, agitation and rolling) through non-covalent bonding [19]. This interaction could increase the amount of bound phenolic compounds in the biscuits. 

In vitro simulated gastrointestinal digestion was performed to evaluate the bio-accessibility of the phenolic compounds in the biscuits. Even though the in vitro model of digestion cannot represent the real digestion in human gastrointestinal tract with limitations to mimic the morphology and anatomical structure of digestion tract and peristaltic movement, it can be a simple and rapid method with no ethical restrictions to be used to analyse how foods being digested by enzymes and the effects of interactions of food ingredients on the release of nutrients. After the gastric stage of in vitro digestion, the bio-accessible fractions had a significantly higher phenolic content than the methanol extracts. The gastric digestion of biscuits partially released phenolic compounds into the supernatant, which had a significantly lower phenolic content (2.31–3.10 mg GAE/g dw) than the biscuit total phenolic content (5.26–5.90 mg GAE/g dw). Digestion with pepsin broke down the protein and disrupted the physical texture of the food, indicating a release of protein bound phenolic compounds or those entrapped in the food macro-structure. The increase in phenolic content after gastric digestion has been reported in many studies, such as wheat-shiitake noodles [24] and *Moringa oleifera* leaf-wheat pasta [32]. Compared with the control the phenolic content of the gastric supernatant, obtained after digestion of all *L. edodes* enriched biscuits (as well as 10–15% *A. auricula* and 5% *T. fuciformis*), was significantly (*p* < 0.05) increased.

The supernatants from the in vitro gastrointestinal digesta had 20–47% greater phenolic content compared to the gastric digesta. During this stage the starch was digested by α-amylase, releasing the phenolic compounds that had been bound to the macromolecules. The inclusion of mushrooms in most substitution levels significantly increased the phenolic content compared with the control biscuit after gastrointestinal digestion (*p* < 0.05), except for 5% *T. fuciformis*. Zieliński, et al. [33] observed a 4-fold increase in phenolic compound content after gastrointestinal digestion of buckwheat biscuits compared with the biscuits before digestion. Phenolic compounds can bind onto starch, protein and dietary fibres, and they are freed from the food matrix under gastrointestinal digestion due to the change of pH (from 2 to 7) and enzymatic hydrolysis of nanoparticles [19,34]. The hydrolysis of those macromolecules and their interactions with the phenolic compounds may positively affect the release of phenolic compounds during digestion. The increase of phenolic content in gastric and intestinal fractions for mushroom enriched biscuits could be that mushroom phenolic compounds are more digestible than sorghum phenolic compounds. 

The phenolic bio-accessibility index after simulated gastric (BI_G_) and intestinal (BI_I_) digestion are shown in Table 1, the BI_G_ of biscuits was 43.99–53.17%, and the BI_I_ values increased to 62.96–90.32%. Compared with the control biscuit the BI_G_ values were significantly increased in several samples, including 10%–15% *L. edodes*, 15% *A. auricula* and 5% *T. fuciformis* enriched biscuits. An increase of BI_I_ value was observed in all levels of *L. edodes* incorporated biscuits, and 15% *A. auricula* and 10–15% *T. fuciformis* enriched biscuits. During the digestion process digestive enzymes, bile salts and pH change all act on the phenolic compounds via processes such as oxidation and hydrolysis, this affects their structure and stability altering their form and thus influencing their bio-accessibility [20,35]. Meng, et al. [36] and Quan, et al. [37] reported that alkaline conditions in the intestinal tract can degrade the phenolic compounds of fruit during in vitro digestion. The BI_I_ values of *T.* *fuciformis* biscuits (10% and 15%) almost reached 100%, however, there was an abundance of phenolic compounds retained in the undigested residues (Table 2). It should be noted that the bio-accessibility index could be affected by the limitations of the Folin-Ciocalteu assay. The Folin reagent may react with some fatty acids, Fe^2+^ ions, free amino acids and peptides released from the food matrix [33,38] which would result in the overestimation of the phenolic content of the bioavailable fractions. The alkaline extraction of bound phenolic compounds, however, (as described in 2.5) may destroy some phenolic compounds, resulting in their loss and subsequent under estimation. Insoluble dietary fibre (Table S1) can be resistant to the release of phenolic compounds even under alkaline hydrolysis which could affect the mushroom-enriched biscuits as they have a high insoluble dietary fibre content. Some previous studies determined the content of phenolic compounds by the Folin-Ciocalteu assay and calculated bio-accessibility after in vitro gastrointestinal digestion of cereal products (Ketnawa, Suwannachot, & Ogawa, 2020; Wang et al., 2020; Zieliński, Szawara-Nowak, & Wronkowska, 2020). However, their calculations for the bio-accessibility were based on methanol extraction only and did not take into consideration the bound phenolic compounds. For instance, Zieliński, Szawara-Nowak and Wronkowska [33] found more than three times of increase of bioavailable phenolic content than the methanol extracts of buckwheat biscuits, and they reported a bio-accessibility index of over 300%. In contrast, Blanco Canalis, Baroni, Leon and Ribotta [21] found a lower phenolic content in the in vitro digested bio-accessible fractions than in the acetone-water extracts of peach puree enriched wheat cookies.

### 3.2. Protein Profile and Soluble Protein Content after Digestion

**Figure 1:**
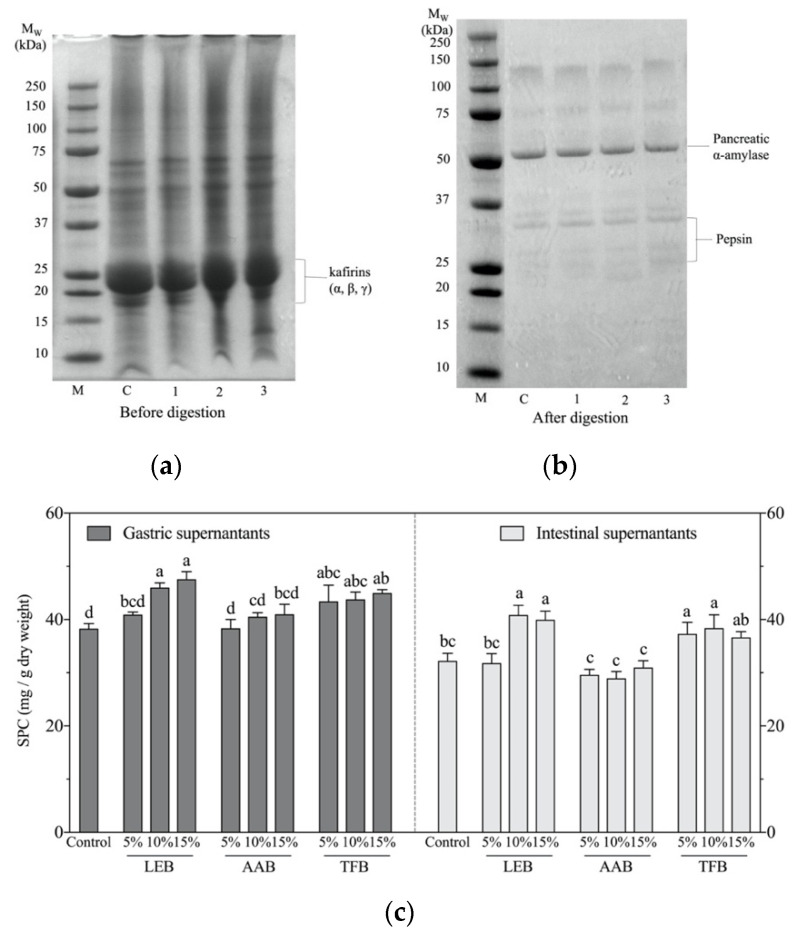
SDS-page analysis of sorghum biscuits enriched with mushroom powder (15% substitution) (**a**) before and (**b**) after gastrointestinal digestion. (**c**) Soluble protein content (SPC) of biscuits after in vitro gastrointestinal digestion. (M—marker; C—control biscuit; 1—15% *L. edodes* biscuit; 2—15% *A. auricula* biscuit; 3—15% *T. fuciformis* biscuit). Error bars represent standard deviation (*n* = 3). Columns with different letters are significantly different within the same chart (*p* < 0.05). LEB—*L. edodes* biscuit; AAB—*A. auricula* biscuit; TFB—*T. fuciformis* biscuit.

The incorporation of *L. edodes* mushroom (5–15%) and *A. auricula* (15%) mushroom significantly increased the protein content of sorghum biscuits (Table S1). However, there was no significant difference between *T. fuciformis* enriched biscuits and the control biscuits. The protein profile distribution of the biscuits, with 15% mushroom enrichment and their digests were analysed using SDS-page under reducing conditions (Figure 1a,b). Before digestion, the pattern of proteins in biscuits displayed major bands with a molecular weight between 18–28 kDa, corresponding to kafirins [5]. The non-kafirin fractions were storage proteins globulin-1 (~65 kDa) and granule-bound starch synthase 1 (~50 kDa) [39]. Sorghum kafirins can be classified into three main fractions according to their molecular weight in α-kafirins (25 and 23 kDa), β-kafirins (20, 18 and 16 kDa) and γ-kafirins (28 kDa) [7]. The sorghum biscuits showed a high-intensity band at ~23 kDa (α-kafirins). The protein profile of 15% *T. fuciformis* had a small band at ~12 kDa, showing an increase in the small *Mw* protein fractions. After the complete gastrointestinal digestion, an overall decline or even disappearance of the band was observed due to enzymatic hydrolysis and proteolysis of the proteins, that produced small peptides (<10 kDa), oligopeptides and free amino acids which are not retained in the gels. The distinct bands (~25–50 kDa) that appeared on the gels of the digesta correspond to pancreatin and pepsin, this was also found in previous studies [40,41].

*L. edodes* digesta supernatant had a higher (*p* < 0.05) soluble protein content than the supernatant of sorghum flour in both gastric and intestinal fractions, while *A. auricula* and *T. fuciformis* had a lower soluble protein content (Figure S2). The soluble protein content of the gastric and intestinal supernatant of the biscuits is shown in Figure 1c. The initial pepsin hydrolysis of sorghum and mushroom proteins in the gastric stage produced large peptides and few of any small peptides or free amino acids. The intestinal stage was crucial for producing oligopeptides and free amino acids [42]. The BCA protein assay used in this study identified the soluble peptides and proteins with three or more amino acid residues. The gastric fractions had a higher soluble protein content than the intestinal fractions, as large polypeptides are hydrolysed, by the enzymes in the pancreatin, into free amino acids or dipeptides and these are not detected by the BCA reagent. The soluble proteins in the intestinal supernatants are not present in the SDS-gels, indicating that these hydrolysed protein fractions are small peptides (<10 kDa) and oligopeptides (short-chain peptides). These small oligopeptides show antioxidant, anti-inflammatory, anticancer, hypocholesterolemic and antihypertensive activities and are likely to be readily absorbed by the intestinal wall [43,44]. Further studies are needed to identify the structure-related absorption and function of the oligopeptides derived from the biscuits after in vitro gastrointestinal digestion.

The soluble protein content of gastric and intestinal supernatant of *L. edodes* and *T. fuciformis* enriched biscuits was higher than the control biscuit (*p* < 0.05), except for 5% *L. edodes* biscuit. The soluble protein content of 10% and 15% *A. auricula* biscuit was higher than the control in gastric fraction, but in intestinal supernatant it was not significantly different to the control. Kafirins, the main protein in sorghum, are proline-rich chains with low water solubility and low enzyme accessibility [5]. While peptide bonds that contain proline cannot be hydrolysed by pancreatic enzymes [42], their digestion and the release of proteins can be influenced by other components in the food matrix such as fibre. Mushrooms are rich in fibre and adding powdered mushrooms may improve protein hydrolysis via the effects of fibre on the physical structure of the biscuit. Ashwath Kumar, et al. [45] discovered that fibre enriched wheat biscuits (TDF, 9.09%) had a higher rate of protein hydrolysis. Sciarini, et al. [46] found that the addition of oat bran fibre and resistant starch into a gluten-free bread (rice flour) increased the percentage of protein hydrolysis and suggested that this was achieved by disrupting the crumb structure. Fibre can also act as a physical barrier to some enzymes and delay the hydrolysis of proteins or polypeptides [18]. The solubility and digestion of protein could also be related to the molecular weight of the protein, and the 15% *T. fuciformis* biscuit had a low *Mw* of protein fraction and a higher soluble protein content compared to the control. Phenolic compounds have been reported to inhibit various digestion enzymes [31,47], by interacting with hydrolysis enzymes. *L. edodes* and *T. fuciformis* biscuits at both 10% and 15% levels had higher intestinal digesta phenolic content than the control, and this is consistent with the soluble protein content in that fraction. The results indicated that the phenolic compounds released after digestion could impede the protein digestion, resulting in an increase of small peptides and oligopeptides retained in the bioaccessible fractions rather than being digested to free amino acids.

### 3.3. In Vitro Antioxidant Activity after Digestion

The antioxidant activities (FRAP and DPPH) of *L. edodes*, *A. auricula* and *T. fuciformis* and sorghum flour are shown in Figure S2. The *L. edodes* had a significantly higher reducing capacity (FRAP) and free radical scavenging ability (DPPH) than the other mushrooms and the flour both before and after digestion (*p* < 0.05), which was consistent with the phenolic content in each fraction.

**Figure 2:**
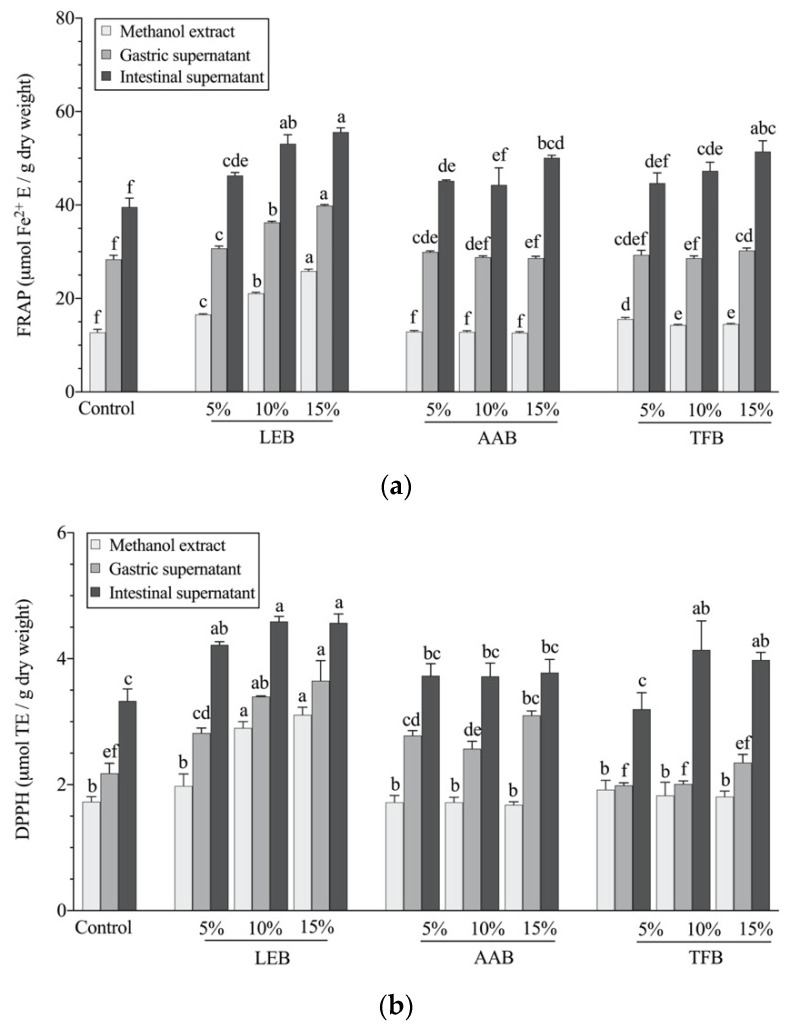
In vitro bio-accessible antioxidant activity of mushroom-enriched sorghum biscuits assessed by Ferrous reducing antioxidant power (FRAP, **a**) and Diphenyl-2-picrylhydrazyl radicals scavenging ability (DPPH, **b**). Values are means ± standard deviation (*n* = 3). The statistical analysis of significance was performed between 10 of the biscuit samples for each fraction (methanol extract, gastric supernatant and intestinal supernatant). Products with different letters are significantly different (*p* < 0.05). LEB—*L. edodes* biscuit; AAB—*A. auricula* biscuit; TFB—*T. fuciformis* biscuit.

The FRAP and DPPH of the sorghum biscuits before and after digestion are shown in Figure 2a,b. It can be seen that *L. edodes* biscuits had a higher reducing capacity (FRAP) and free radical scavenging ability (DPPH) than other biscuits both before and after digestion (*p* < 0.05), which is consistent with the phenolic content of each fraction. The antioxidant activity of the digesta supernatants was significantly higher than the methanol extract of the biscuits, and the activity after intestinal digestion was increased almost three-fold. After in vitro digestion, the physical structure and inter and intra-molecular bonds in the biscuits are hydrolysed by enzymes and the nutrients and antioxidants are released [24]. *A. auricula* and *T. fuciformis* enriched biscuits had a higher content of soluble dietary fibre and this macromolecule could bind to antioxidants in stomach and intestine, delivering the antioxidants to colon. Baczek, et al. [48] found an increase of antioxidant properties (ABTS and FRAP) in the soluble fractions after in vitro digestion of oat-buckwheat bread.

The digestion process, whereby food is exposed to digestive enzymes and variations in pH, is crucial to cause the release of phenolic compounds from the molecules that have bound them [49] The released phenolic compounds are the main contributor to the antioxidant properties [50], however, other molecules associated with the binding or trapping of phenolic compounds can also affect the antioxidant activity. Compared with the control, sorghum biscuits enriched with *L. edodes* had increased the antioxidant activity (FRAP and DPPH) in both gastric and intestinal fractions. *T. fuciformis* 10% and 15% enriched biscuits also had increased (*p* < 0.05) antioxidant activities after digestion. *A. auricula* 15% enriched biscuits had higher FRAP values in the upper gastrointestinal tract than the control samples, but the addition of *A. auricula* did not increase DPPH values. The FRAP assay is based on the reduction of ions from Fe^3+^ to Fe^2+^, and the DPPH reagent can receive hydrogen atoms from antioxidants [20]. That means that the antioxidant compounds assessed by the DPPH are not the same as that of FRAP. The antioxidant activity of food after gastrointestinal digestion is vital for health. For example, antioxidants can scavenge and suppress the excess reactive oxygen species (ROS) in the organisms and prevent oxidative-related diseases [51]. Otherwise, excessive ROS causes inflammation which leads to diseases, such as inflammatory bowel disease.

### 3.4. Phenolic and Antioxidants Content in Undigested Residue

Both the free and bound phenolic content of undigested residue increased with the incorporation of mushrooms, as can be seen in Table 2. Mushroom enrichment in the sorghum biscuits increased the dietary fibre content of biscuits and total phenolic content of the undigested residue meaning that there were more phenolic compounds available to transit into the colon. The phenolic compounds in vegetables or mushrooms are normally conjugated with dietary fibres [15], which explains the increased phenolic content in the undigested residue. Phenolic compounds in the pellets can undergo biological metabolism by colonic microbiota and microbial enzymes [52]. Dong, et al. [53] demonstrated that the bound polyphenols in carrot dietary fibre were liberated during in vitro fermentation, and the fermented polyphenols could promote the growth of specific beneficial flora and suppress the harmful bacterial flora. Fermentation by microbiota can promote the bioavailability and absorption of phenolic compounds. The bio-absorption of polyphenolic metabolites fermented by gut microbiota may reach the liver via the hepatic portal vein after absorption, and undergo further degradation and enter into systematic circulation before reaching targeted tissues and cells [54].

The free phenolic content of the undigested residue and the FRAP activity was decreased in the *A. auricula* biscuits compared to the control and was increased in the *L. edodes* and *T. fuciformis* biscuits. However, the total reducing capacity (FRAP) of the undigested residue increased when the biscuits were enriched with mushroom powder, due to the increase in antioxidant activity of the bound fraction of the undigested residue. The DPPH activity of the undigested residue of mushroom enriched biscuits showed that they had a higher (*p* < 0.05) total radical scavenging capacity than the control biscuits.

### 3.5. β-Glucan Potential Colon-Bioavailability

**Figure 3:**
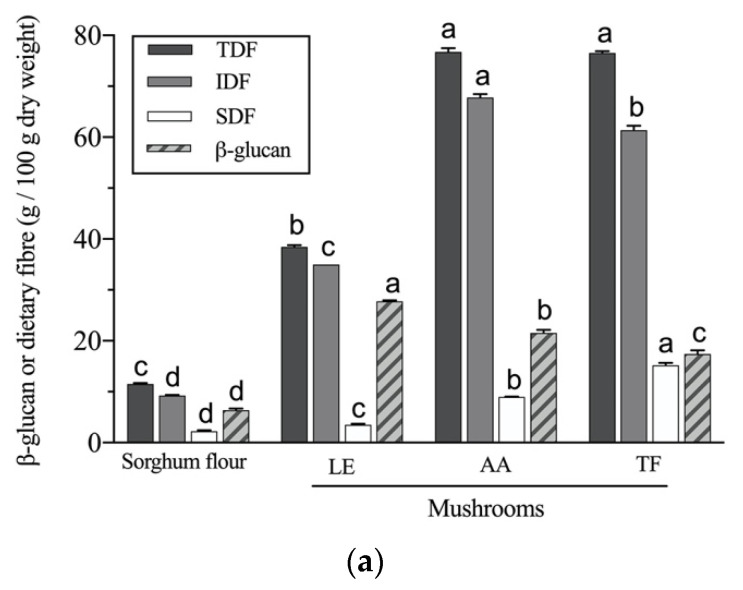

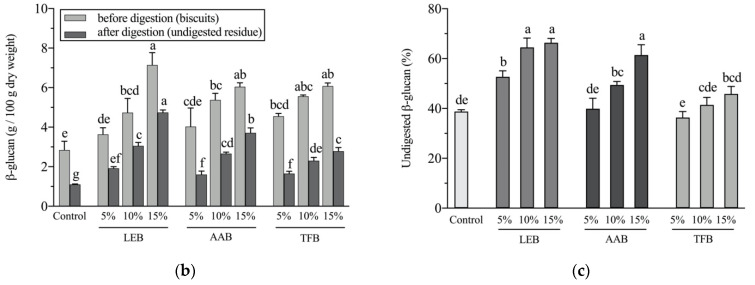
β-glucan and dietary fibre content of sorghum flour and mushrooms (**a**); and β-glucan content of biscuits and undigested residue (g/100 g dry weight biscuit) (**b**); and the undigested β-glucan (%) after gastrointestinal digestion (**c**). Undigested β-glucan (%) was calculated by the following formula: Undigested β-glucan (%) = (gram of β-glucan in the undigested residue/gram of β-glucan in the initial biscuits) × 100. Values = means ± standard deviation (*n* = 3). The statistical analysis of significance was performed between four of the materials for figure (**a**) and between 10 of the biscuit samples for figures (**b**,**c**). Products with different letters are significantly different (*p* < 0.05). LEB—*L. edodes* biscuit; AAB—*A. auricula* biscuit; TFB—*T. fuciformis* biscuit.

β-glucan is abundant in both cereals and mushrooms, and it can bring various disease prevention properties, such as reducing postprandial blood glucose and lowering LDL cholesterol. The sorghum flour contained a total of 6.41 g/100g dw of β-glucan, which was much lower than mushroom samples, as can be seen in Figure 3a. The *L. edodes* had a high content of β-glucan (27.78 g/100g dw), *A. auricula* had slightly less (21.55 g/100g dw) and *T. fuciformis* had least (17.44 g/100g dw), and the results are similar to the β-glucan content of several other mushroom cultivars (9 to 27 g/100g dw) [55]. Cereal β-glucan has a fibrous structure with a combination of 1-3 β-glycosidic and 1-4 β-glycosidic linkages, while mushroom β-glucan mainly consists of 1-3 β-glycosidic with 1-6 β-glycosidic branches. The different sources of β-glucan have diverse molecular structures, such as molecular weight, conformation and branching degree, influencing their solubility, viscosity and rheological characteristics [56]. For example, the chemical structure and molecular weight of β-glucan are two main factors that determine the solubility of β-glucan [57]. The sorghum flour, *L. edodes* and *A. auricula* all had a much lower soluble fibre content than β-glucan content, especially *L. edodes* samples indicating that more β-glucan is water-insoluble in *L. edodes*. Morales, et al. [58] found that *Lentinula edodes* had a low yield (4.2%) of hot water-soluble extract and a high content (27%) of β-glucan, which was consistent with *L. edodes* mushroom used in this study. They further determined the β-glucan of the hot water-soluble extract and the resulting insoluble fraction after extraction, finding that the insoluble fraction had a higher content of β-glucan (38%) than the soluble extract (13.2%). The solubility of β-glucan largely depends on the percentage of 1-3 β-glycosidic linkages, because this type of linkage leads to twists in the straight-chain polymer allowing water molecules to enter into the chains [59]. Lentinan is a water-soluble polysaccharide from *L. edodes* with a primary structure of the (1-3)-β-D-glucan [60]. The solubility might also be influenced by interactions with other macronutrients in mushrooms, such as insoluble fibre and proteins [61]. β-glucan could covalently connect with the chitin (insoluble fibre) [62], leading to a high content of insoluble β-glucan content in mushroom samples. Alahmed and Simsek [57] found a decrease in the solubility of oat β-glucan due to the increase of molecular weight because of a rise in cohesive energy density. The molecular weight of β-glucan also relates to its viscosity and thus affects its functionalities (such as hypoglycaemic and hypocholesterolemic properties) [56].

The addition of powder of any of the three mushroom species used in this study significantly (*p* < 0.05) increased the β-glucan contents of sorghum biscuits and their undigested residues (Figure 3b). After in vitro digestion, β-glucan content was higher in the undigested residue than in the original biscuits showing that it is mostly indigestible, especially in *L. edodes*-enriched samples. The dietary fibre (non-digestible polysaccharides) and the nutrients, phenols and other bioactive compounds that are bound to them that remain after gastric and intestinal digestion, this remaining portion is a prediction of composition of the digesta that would pass into the colon. The solubility of β-glucan during the in vitro digestion process affects the precipitation of β-glucan, and the intestinal digestion condition (pH 7) may cause the β-glucan to aggregate [52]. The undigested residue of *L. edodes* enriched biscuits had an increased β-glucan (1.92–4.74 g/100g dw biscuit) compared to the control (1.10 g/100g dw biscuit). Significantly higher content was also found in the undigested residues of the *A. auricula* enriched biscuits (1.61–3.71 g/100g dw biscuit) and *T. fuciformis* enriched biscuits (1.66–2.79 g/100g dw biscuit). The expression ‘’undigested β-glucan’’ (%) was used to indicate the percentage of β-glucan from the biscuits that would potentially be available for fermentation by the colonic microbiota. As shown in Figure 3c, the undigested β-glucan (%) increased significantly in mushroom enriched biscuits after in vitro gastrointestinal digestion, except for 5% *A. auricula* and 5% *T. fuciformis*. The control biscuit had a 38.75% of undigested β-glucan, and the percentage increased to 66.36% in 15% *L. edodes biscuit* as well as 61.44% in 15% *A. auricula biscuit*. There was only a 7.11% increase in 15% *T. fuciformis* biscuit. 

The β-glucan that is undigested by the stomach or intestine acts as a conveyer of phenolic compounds to the colon, it does this by forming a gel network, which limits the solubilisation of phenolic compounds in stomach and intestine [52]. Another important function of β-glucan in the colon is to be fermented and utilised by gut microbiota. In vivo studies have indicated that consuming mushroom β-glucan or polysaccharides causes an increase in the production of short-chain fatty acids (SCFAs) as a result of fermentation by colonic microbiota; this in return, modulates the gut flora community and regulates inflammatory bowel diseases [61,63,64]. 

### 3.6. Principal Component Analysis and Correlations

**Figure 4:**
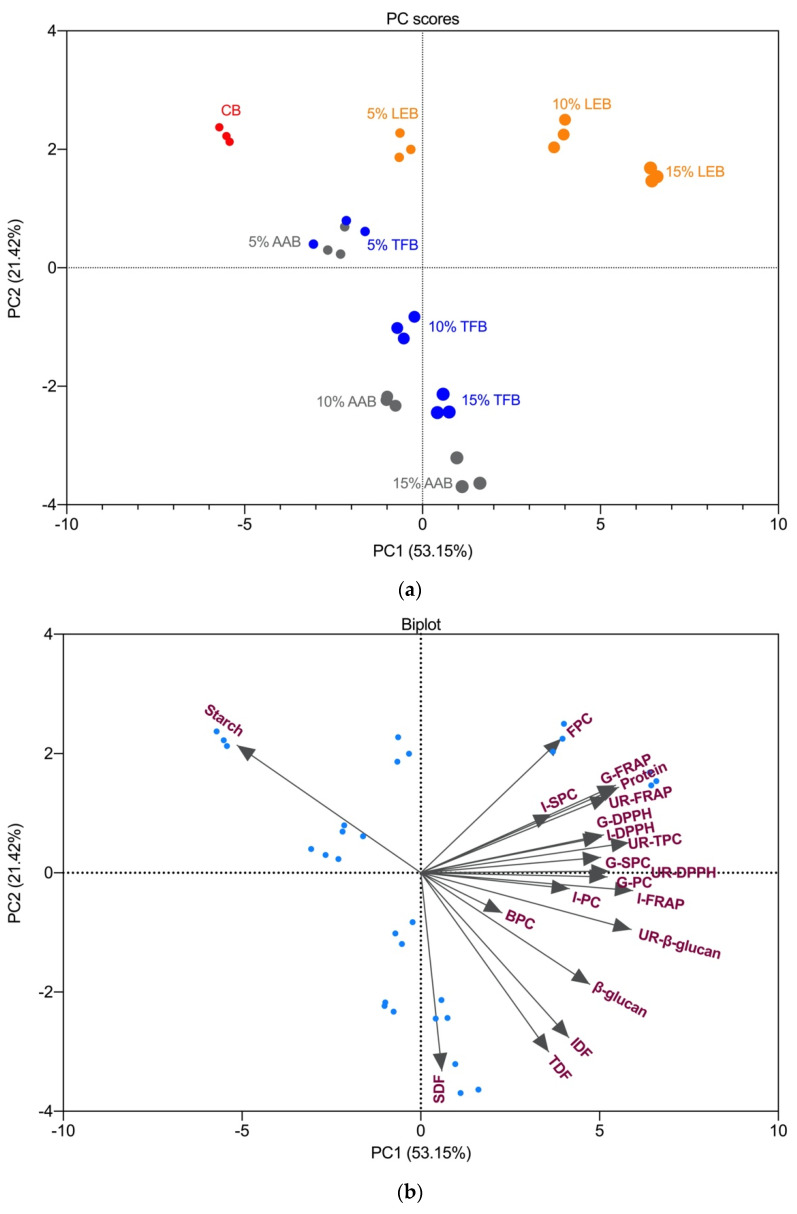
Principal component analysis of principal component scores biplot. PC scores distribution of different biscuit samples incorporated with dried mushrooms (**a**) and Biplot component loading (**b**) as obtained from the principal component analysis. Abbreviations: CB—control biscuits; LEB—*L. edodes* biscuit; AAB—*A. auricula* biscuit; TFB—*T. fuciformis* biscuit; FPC—free phenolic content biscuits; BPC—bound phenolic content of biscuits; G-PC, G-FRAP and G-DPPH represent the phenolic content and antioxidant activity of gastric supernatant; I-PC, I-FRAP and I-DPPH, represented the phenolic content and antioxidant activity of intestinal supernatant; G-SPC—soluble protein content of gastric supernatant; I-SPC—soluble protein content of intestinal supernatant; TDF—total dietary fibre; SDF—soluble dietary fibre; IDF—insoluble dietary fibre; UR-TPC, UR-FRAP and UR-DPPH represented the total phenolic content and total antioxidant activity of the undigested residue; UR-β-glucan—β-glucan content of undigested residue.

The principal component 1 (PC1) explained 53.15% of the total variance, while the PC2 accounts for the subsequent 21.42% of the total variance. In this case, Figure 4 explains 74.57% of the variability, which shows that PC1 and PC2 both had a large contribution to explain the response variables. The control biscuit was loaded in the negative axis of PC1 and the positive axis of PC2 (Figure 4a). The control biscuit was positively related to starch content, and more negatively related to insoluble dietary fibre, total dietary fibre, β-glucan, undigested residue β-glucan, bound phenolic content and intestinal digesta phenolic content. PC1 highly discriminated the *L. edodes* enriched biscuits from the control biscuit in terms of the increased substitution levels. *L. edodes* incorporation was more positively related to the free phenolic content, protein, soluble protein content of intestinal supernatant, and antioxidant activity of the bio-accessible fractions. The score of the *A. auricula* and *T. fuciformis* enriched biscuits moved downward with increasing substitution levels. That means that the enrichment with *A. auricula* and *T. fuciformis* was mainly characterised by high dietary fibre, β-glucan and β-glucan of undigested residue content and a high bound phenolic content. Overall, the results analysed by the PCA model illustrated that the enrichment with mushroom powders had a significant effect on the parameters of the sorghum biscuits that were analysed. 

Pearson’s correlation was conducted to evaluate the correlations between the nutritional composition, bio-accessible phenolic content, peptides, antioxidants and β-glucan remaining after in vitro digestion. The protein content of biscuits was positively correlated with the soluble protein content of gastric supernatant (*r* = 0.662, *p* < 0.001) and intestinal supernatant (*r* = 0.464, *p* < 0.01) (Table S2). This means that the biscuits with a higher protein content had a higher soluble peptide content in the in vitro digested supernatant. The phenolic content of both gastric and intestinal supernatant was positively correlated (*p* < 0.001) with the antioxidant activities (FRAP and DPPH). There was a positive correlation between soluble protein content and phenolic content of gastric supernatant (*r* = 0.601, *p* < 0.001). A similar correlation was observed in the intestinal supernatant (*r* = 0.594, *p* = 0.001). These findings were added of interest and suggested that the soluble peptides in the digest supernatants might interact with phenolic compounds and potentially help transport the bioactive compounds for further intestinal uptake.

Insoluble dietary fibre content was positively correlated with the phenolic content of both gastric and intestinal supernatant and antioxidant activity of the intestinal supernatant. The results showed that there were positive correlations between biscuit insoluble dietary fibre and gastric digesta phenolic content (*r* = 0.524, *p* < 0.01), intestinal digesta phenolic content (*r* = 0.463, *p* = 0.01), intestinal digesta FRAP (*r* = 0.634, *p* < 0.001) and intestinal digesta DPPH (*r* = 0.362, *p* < 0.05). The soluble dietary fibre did not show significant correlations with the intestinal digesta phenolic and antioxidant content. The explanation could be that insoluble dietary fibre has a higher phenolic and antioxidant content than soluble dietary fibre [15]. 

Mushroom enrichment improved the total insoluble dietary fibre content of sorghum biscuits. Part of the phenolic compounds and antioxidants contained in insoluble dietary fibre were released during the gastrointestinal digestion of biscuits. Phenolic content of gastric and intestinal supernatant from mushroom enriched biscuits were increased. Phenolic compounds trapped in mushroom dietary fibre could be easier to be released than sorghum insoluble fibre during digestion. It might be due to the difference in the structure of insoluble fibre between mushroom (chitin-β-glucan) and sorghum (cellulose). One recent study compared the release of phenolic compounds from different cereal sources of insoluble dietary fibre (wheat, barley, quinoa and triticale) under in vitro simulated gastrointestinal digestion and found that insoluble fibre from quinoa and triticale had a significantly higher phenolic content in both gastric and intestinal fractions than wheat and barley [65]. 

Dietary fibres were reported to have negative effects on the release and absorption of phenolic compounds by their molecular interactions [66]. A positive correlation was found between β-glucan content of the undigested residue and its total phenolic content (*r* = 0.754, *p* < 0.001), FRAP (*r* = 0.588, *p* = 0.001) and DPPH (*r* = 0.706, *p* < 0.001). Insoluble dietary fibre had a lower correlation coefficient (*r* = 0.456, *p* < 0.05) with total phenolic content of the undigested residue. The β-glucan remaining might be the main insoluble fibre that contributes to the potential delivery of bioactive compounds to the colon. The enriched mushroom insoluble dietary fibre might have a higher content of bound phenolic compounds than sorghum insoluble fibre. Insoluble dietary fibre also favoured the accumulation of β-glucan in the colon as a positive correlation was found between insoluble dietary fibre and β-glucan of undigested residue (*r* = 0.788, *p* < 0.001).

### 3.7. Nutritional Value

Industrial production of biscuits would require the information of the functional ingredients in the manipulation of the quality and nutritional value of the final products. The optical addition of mushroom powder into cereal products were between 5% and 15% with minor or no negative effects on the sensory acceptability and slight changes in physical characteristics [16]. In the nutritional view of point, enrichment of sorghum biscuits with mushrooms enhanced the dietary fibre and β-glucan content. Previous study has illustrated a close association between an increase of dietary fibre consumption and a lower incidence of obesity and type-2 diabetes in relation to gut microbiota [67]. Daily consumption of 3 g of β-glucan is recommended to have a healthy indicate of cholesterol-lowering [68]. This means that a serving of approximately 50 g of biscuits enriched with 15% mushroom can satisfy this recommendation. Glycaemic carbohydrate is normally high in biscuits, but mushroom fortification reduced the in vitro glycaemic glucose value (Table S2), especially for 15% *A. auricula* and *T. fuciformis* enriched sample with a lower than 30 g glucose per 100 g dry weight. The high fibre content and low glucose level of biscuits could be considered to be diabetic-friendly. The bioavailable small peptides and antioxidants was improved by mushroom enrichment, which can be linked to help relief of chronic diseases. Apart from being nutrient-rich, sorghum biscuits are gluten-free that offers significant opportunities for those population with coeliac disease. These properties added the interest of developing functional products in the future.

## 4. Conclusions

Enrichment of sorghum biscuits with mushroom powders improved their nutritional quality. Most of mushroom-containing biscuits had a higher content of phenolic compounds quantified in their in vitro digested supernatants and better antioxidant activity than the control biscuit, indicating that mushroom fortification enhanced the bioavailable phenolic content. The digested *L. edodes* and *T. fuciformis* biscuits contained more soluble peptides than the control and *A. auricula* biscuits. The soluble peptides had a small molecular weight that cannot be identified by the SDS-page gel. The undigested residue of mushroom enriched biscuits had a higher remaining portions of phenolic compound and β-glucan than the control, which was related to the insoluble dietary fibre. These findings provide an understanding of the nutritional and functional benefits of mushroom enriched biscuits under the in vitro gastrointestinal digestion and support the idea that mushroom enrichment can potentially increase the bio-accessible phenolic content and the proportions of phenolic compounds and β-glucan delivered to colon. This means the developed biscuits would have a real application in the future with a potential to attenuate chronic diseases. Future experiments are required to evaluate the functionalities of the biscuits ingredients released or remained during digestion through modelled cell line and in vivo studies.

## Figures and Tables

**Table 1:** The phenolic content of sorghum flour, mushrooms and mushroom-enriched sorghum biscuits. Phenolic content of the digesta supernatant after gastric and intestinal in vitro digestion, and the bio-accessibility index of the phenolic compounds in those digesta. Different uppercase letters represent significant difference of values between the ingredients (*p* < 0.05), while the lowercase letters represent significant difference of values between the biscuits (*p* < 0.05).

Samples	FPC	BPC	TPC	Gastric Fractions	Intestinal Fractions	Bio-Accessibility Index (%)	
						BI_G_	BI_I_
Ingredients							
*Sorghum*	2.98 ± 0.18 ^B^	7.49 ± 0.14 ^A^	10.47 ± 0.30 ^B^	3.89 ± 0.01 ^C^	6.45 ± 0.06 ^C^	37.23 ± 1.11 ^C^	61.69 ± 2.14 ^C^
*L. edodes*	7.16 ± 0.11 ^A^	7.47 ± 0.22 ^A^	14.63 ± 0.24 ^A^	9.55 ± 0.08 ^A^	13.36 ± 0.38 ^A^	65.26 ± 0.64 ^A^	91.33 ± 1.30 ^A^
*A. auricula*	1.37 ± 0.07 ^C^	7.30 ± 0.17 ^A^	8.67 ± 0.10 ^C^	4.80 ± 0.22 ^B^	6.93 ± 0.28 ^BC^	55.29 ± 2.47 ^B^	79.87 ± 4.00 ^B^
*T. fuciformis*	1.23 ± 0.07 ^C^	6.65 ± 0.03 ^B^	7.89 ± 0.06 ^D^	4.58 ± 0.11 ^B^	7.50 ± 0.12 ^B^	58.08 ± 1.75 ^B^	95.08 ± 1.19 ^A^
Biscuits							
Control	1.78 ± 0.01 ^c^	3.48 ± 0.04 ^e^	5.26 ± 0.03 ^e^	2.31 ± 0.12 ^e^	3.36± 0.10 ^e^	43.99 ± 2.51 ^d^	63.84 ± 2.27 ^e^
5% LEB	1.79 ± 0.01 ^c^	3.68 ± 0.05 ^bcd^	5.48 ± 0.04 ^d^	2.44 ± 0.05 ^de^	4.09 ± 0.18 ^c^	44.58 ± 0.69 ^d^	74.71 ± 2.91 ^bc^
10% LEB	1.94 ± 0.03 ^b^	3.74 ± 0.03 ^bc^	5.68 ± 0.06 ^bc^	2.98 ± 0.06 ^ab^	4.48 ± 0.07 ^b^	52.42 ± 1.08 ^abc^	78.82 ± 0.67 ^b^
15% LEB	2.08 ± 0.03 ^a^	3.82 ± 0.02 ^ab^	5.90 ± 0.04 ^a^	3.10 ± 0.05 ^a^	4.53 ± 0.09 ^ab^	52.65 ± 1.11 ^ab^	76.88 ± 1.20 ^b^
5% AAB	1.75 ± 0.02 ^cd^	3.95 ± 0.02 ^a^	5.70 ± 0.04 ^b^	2.54 ± 0.10 ^cde^	3.87 ± 0.03 ^cd^	44.50 ± 1.93 ^d^	67.83 ± 0.57 ^de^
10% AAB	1.67 ± 0.02 ^ef^	3.81 ± 0.03 ^ab^	5.48 ± 0.05 ^d^	2.60 ± 0.05 ^cd^	3.61 ± 0.06 ^de^	47.51 ± 0.92 ^cd^	65.87 ± 1.58 ^de^
15% AAB	1.62 ± 0.02 ^f^	3.85 ± 0.15 ^ab^	5.47 ± 0.16 ^d^	2.91 ± 0.13 ^ab^	3.85 ± 0.12 ^cd^	53.17 ± 2.28 ^a^	70.28 ± 2.44 ^cd^
5% TFB	1.79 ± 0.02 ^c^	3.70 ± 0.04 ^bcd^	5.49 ± 0.04 ^cd^	2.78 ± 0.03 ^bc^	3.46 ± 0.08 ^e^	50.57 ± 0.80 ^abc^	62.96 ± 1.71 ^e^
10% TFB	1.74 ± 0.03 ^cd^	3.61 ± 0.04 ^cde^	5.35 ± 0.04 ^de^	2.55 ± 0.13 ^cde^	4.83 ± 0.06 ^a^	47.67 ± 2.58 ^cd^	90.32 ± 1.27 ^a^
15% TFB	1.70 ± 0.02 ^de^	3.56 ± 0.07 ^de^	5.26 ± 0.06 ^e^	2.52 ± 0.12 ^de^	4.70 ± 0.21 ^ab^	47.78 ± 1.73 ^bcd^	89.37 ± 2.94 ^a^

Values = means ± standard deviation (*n* = 3). FPC—free phenolic content (methanol extraction); BPC—bound phenolic content (alkaline hydrolysis); TPC—total phenolic content; TPC = FPC + BPC. Values in the same column for ingredients with different uppercase letters are significantly different (*p* < 0.05). Values in the same column for biscuits with different lowercase letters are significantly different (*p* < 0.05). Abbreviations: LEB- *L. edodes* biscuit; AAB—*A. auricula* biscuit; TFB—*T. fuciformis* biscuit; BI (bio-accessibility index) = phenolic content of gastric (or intestinal) supernatant/total phenolic content of biscuit.

**Table 2:** Phenolic content and potential antioxidant activity of the undigested residue after in vitro gastrointestinal digestion. Different letters represent significant difference of values between the biscuits (*p* < 0.05).

Samples	Phenolic Content (mg GAE/g dw)			FRAP (μmol Fe^2+^ E/g dw)			DPPH (μmol TE/g dw)		
	Free	Bound	Total	Free	Bound	Total	Free	Bound	Total
Control biscuit	0.58 ± 0.01 ^e^	0.56 ± 0.01 ^f^	1.15 ± 0.01 ^f^	6.34 ± 0.41 ^d^	8.41 ± 0.17 ^d^	14.75 ± 0.46 ^e^	0.95 ± 0.00 ^e^	0.91 ± 0.06 ^c^	1.85 ± 0.06 ^e^
5% LEB	0.78 ± 0.01 ^b^	0.90 ± 0.02 ^ab^	1.67 ± 0.03 ^b^	7.04 ± 0.17 ^ab^	12.59 ± 0.38 ^a^	19.62 ± 0.45 ^a^	1.07± 0.01 ^ab^	1.05 ± 0.03 ^ab^	2.12 ± 0.03 ^abcd^
10% LEB	0.84 ± 0.01 ^a^	0.94 ± 0.05 ^a^	1.78 ± 0.05 ^a^	7.05 ± 0.20 ^ab^	13.19 ± 0.41 ^a^	20.24 ± 0.55 ^a^	1.09 ± 0.02 ^a^	1.14 ± 0.06 ^a^	2.23 ± 0.06 ^ab^
15% LEB	0.85 ± 0.02 ^a^	0.96 ± 0.03 ^a^	1.77 ± 0.06 ^a^	7.31 ± 0.07 ^a^	12.91 ± 0.10 ^a^	20.22 ± 0.04 ^a^	1.09 ± 0.00 ^a^	1.15 ± 0.02 ^a^	2.24 ± 0.02 ^a^
5% AAB	0.60 ± 0.01 ^e^	0.77 ± 0.02 ^cde^	1.37 ± 0.02 ^de^	5.27 ± 0.13 ^e^	9.92 ± 0.26 ^c^	15.20 ± 0.37 ^de^	0.93 ± 0.02 ^e^	1.06 ± 0.01 ^ab^	1.99 ± 0.02 ^de^
10% AAB	0.65 ± 0.01 ^d^	0.79 ± 0.02 ^cd^	1.45 ± 0.01 ^d^	5.30 ± 0.09 ^e^	10.18 ± 0.15 ^bc^	15.49 ± 0.24 ^cde^	1.04 ± 0.01 ^bc^	1.10 ± 0.04 ^ab^	2.13 ± 0.04 ^abc^
15% AAB	0.72 ± 0.02 ^c^	0.84 ± 0.03 ^bc^	1.56 ± 0.03 ^c^	5.64 ± 0.08 ^e^	10.99 ± 0.16 ^b^	16.63 ± 0.24 ^bc^	1.04 ± 0.03 ^bc^	1.10 ± 0.05 ^ab^	2.14± 0.07 ^abc^
5% TFB	0.59 ± 0.01 ^e^	0.71 ± 0.01 ^e^	1.30 ± 0.01 ^e^	6.41 ± 0.06 ^cd^	9.61 ± 0.69 ^c^	16.02 ± 0.75 ^bcd^	1.00 ± 0.01 ^cd^	1.10 ± 0.02 ^ab^	2.10 ± 0.03 ^bcd^
10% TFB	0.67 ± 0.01 ^d^	0.72 ± 0.03 ^de^	1.39 ± 0.02 ^de^	6.70 ± 0.07 ^bcd^	10.00 ± 0.17 ^bc^	16.70 ± 0.24 ^b^	0.99 ± 0.01 ^d^	1.00 ± 0.01 ^bc^	2.00 ± 0.01 ^d^
15% TFB	0.70 ± 0.04 ^cd^	0.75 ± 0.03 ^de^	1.45 ± 0.04 ^d^	6.85 ± 0.12 ^abc^	10.07 ± 0.47 ^bc^	16.91 ± 0.39 ^b^	0.99 ± 0.01 ^d^	1.04 ± 0.06 ^ab^	2.02 ± 0.07 ^cd^

Values = means ± standard deviation (*n* = 3). Values in the same column with different letters are significantly different (*p* < 0.05). Total = Free + Bound for phenolic content, FRAP and DPPH. LEB—*L. edodes* biscuit; AAB—*A. auricula* biscuit; TFB—*T. fuciformis* biscuit.

## Supplementary Materials

The following are available online at https://www.mdpi.com/article/10.3390/foods10081812/s1, Table S1: Formula of sorghum biscuits enriched with dried mushroom powders, Table S2: Starch, protein and dietary fibre contents and in vitro glycaemic response of sorghum biscuits enriched with dried mushroom powders, Figure S1: Soluble protein content (SPC) of mushrooms and sorghum flour after in vitro gastrointestinal digestion., Figure S2: In vitro bioaccessible antioxidant activity of mushrooms and sorghum flour assessed by FRAP (a) and DPPH (b). Values are means ± standard deviation (*n* = 3). Products with different letters are significantly different (*p* < 0.05). Figure S3: Pearson’s correlations between the observed biscuits parameters before and after in vitro digestion.
