# Supplementary material for: Delivery of Phenolic Compounds, Peptides and β-Glucan to the Gastrointestinal Tract by Incorporating Dietary Fibre-Rich Mushrooms into Sorghum Biscuits
Source: Foods. 2021 Aug 5;10(8):1812. doi: 10.3390/foods10081812 (PMC8391148; doi:10.3390/foods10081812)
Supplement: Supplementary file 1 [file foods-10-01812-s001.zip › foods-1296021-supplementary.pdf]

foods

Supplementary Materials:

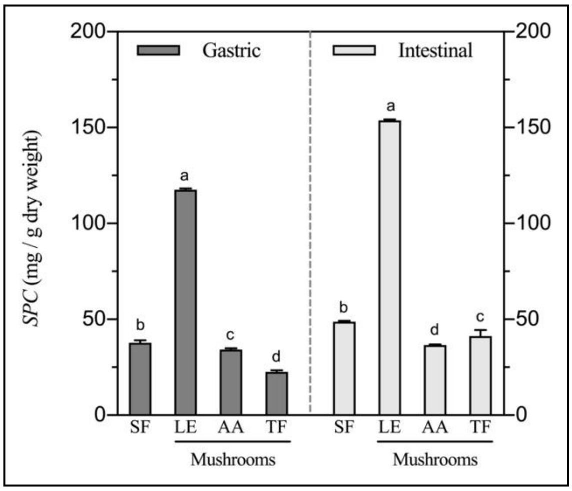

Figure S1. Soluble protein content (SPC) of mushrooms and sorghum flour after *in vitro* gastrointestinal digestion. Values are means ± SD of triplicates. Means between different products with different letters are significantly different ($p < 0.05$).

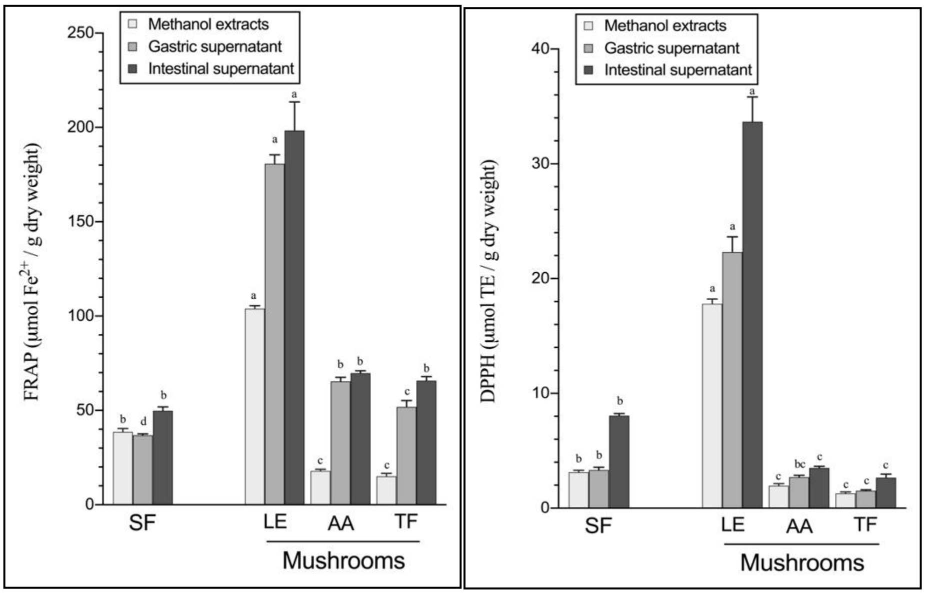

(**a**) (**b**)

Figure S2. *In vitro* bioaccessible antioxidant activity of mushrooms and sorghum flour assessed by FRAP (**a**) and DPPH (**b**). Values are means ± SD of triplicates. Means between different products with different letters are significantly different ($p < 0.05$).

	TDF	SDF	IDF	G-SPC	G-PC	G-FRAP	G-DPPH	I-SPC	I-PC	I-FRAP	I-DPPH	Protein	Starch	β-glucan	UR-β-glucan	UR-TPC	UR-FRAP	UR-DPPH	FPC	BPC
TDF		0.000	0.000	0.032	0.014	0.696	0.203	0.627	0.012	0.001	0.127	0.634	0.000	0.000	0.000	0.053	0.539	0.018	0.175	0.114
SDF	0.830		0.000	0.382	0.880	0.038	0.156	0.916	0.092	0.222	0.675	0.032	0.001	0.001	0.147	0.558	0.193	0.957	0.000	0.776
IDF	0.990	0.743		0.018	0.003	0.328	0.055	0.542	0.010	0.000	0.049	0.272	0.000	0.000	0.000	0.011	0.286	0.003	0.419	0.046
G-SPC	0.392	0.165	0.428		0.000	0.000	0.053	0.000	0.000	0.000	0.000	0.000	0.116	0.001	0.000	0.001	0.000	0.001	0.001	0.613
G-PC	0.443	0.029	0.524	0.601		0.000	0.000	0.016	0.172	0.000	0.019	0.000	0.006	0.000	0.000	0.000	0.002	0.000	0.003	0.011
G-FRAP	0.074	-0.380	0.185	0.685	0.704		0.000	0.000	0.012	0.000	0.000	0.000	0.262	0.018	0.000	0.000	0.000	0.000	0.000	0.215
G-DPPH	0.239	-0.266	0.354	0.357	0.660	0.774		0.382	0.165	0.000	0.000	0.000	0.003	0.060	0.000	0.000	0.000	0.000	0.001	0.002
I-SPC	0.092	-0.020	0.116	0.822	0.435	0.601	0.165		0.001	0.001	0.006	0.001	0.763	0.051	0.040	0.036	0.001	0.074	0.000	0.186
I-PC	0.452	0.313	0.463	0.639	0.256	0.455	0.260	0.594		0.000	0.000	0.010	0.128	0.002	0.006	0.002	0.001	0.136	0.087	0.665
I-FRAP	0.577	0.230	0.634	0.763	0.736	0.749	0.665	0.555	0.995		0.000	0.000	0.002	0.000	0.000	0.000	0.000	0.000	0.005	0.227
I-DPPH	0.285	-0.080	0.362	0.601	0.427	0.699	0.631	0.489	0.725	0.673		0.000	0.051	0.028	0.001	0.000	0.000	0.001	0.001	0.348
Protein	0.091	-0.392	0.207	0.642	0.662	0.963	0.814	0.557	0.464	0.710	0.755		0.169	0.020	0.000	0.000	0.000	0.000	0.000	0.221
Starch	-0.891	-0.575	-0.923	-0.293	-0.494	-0.212	-0.521	0.058	-0.284	-0.550	-0.359	-0.258		0.000	0.000	0.006	0.275	0.002	0.426	0.012
β-glucan	0.834	0.555	0.860	0.596	0.620	0.427	0.347	0.359	0.539	0.713	0.401	0.423	-0.730		0.000	0.007	0.101	0.006	0.362	0.164
UR-β-glucan	0.714	0.271	0.788	0.635	0.762	0.678	0.726	0.376	0.491	0.828	0.595	0.713	-0.797	0.834		0.000	0.001	0.000	0.024	0.036
UR-TPC	0.357	-0.111	0.456	0.595	0.660	0.783	0.826	0.385	0.536	0.788	0.824	0.851	-0.493	0.485	0.754		0.000	0.000	0.001	0.040
UR-FRAP	0.117	-0.245	0.201	0.664	0.545	0.798	0.690	0.568	0.578	0.720	0.795	0.857	-0.206	0.305	0.588	0.908		0.000	0.000	0.589
UR-DPPH	0.431	-0.010	0.519	0.562	0.782	0.645	0.699	0.331	0.279	0.656	0.583	0.676	-0.544	0.489	0.706	0.814	0.722		0.015	0.003
FPC	-0.254	-0.603	-0.153	0.571	0.524	0.911	0.557	0.613	0.318	0.498	0.556	0.872	0.151	0.173	0.410	0.567	0.696	0.442		0.690
BPC	0.295	-0.054	0.367	-0.096	0.460	0.233	0.552	-0.248	-0.082	0.227	0.177	0.230	-0.455	0.261	0.385	0.376	0.103	0.517	0.076	

Figure S3. Pearson's correlations between the observed biscuits parameters before and after digestion. The right side of the green part is *p* values, whilst the left side of the yellow part is *r* values. CB- control biscuit; LEB- *Lentinula edodes* biscuit; AAB- *Auricularia auricula* biscuit; TFB- *Tremella fuciformis* biscuit; FPC- free phenolic content of biscuits; BPC- bound phenolic content of biscuits; G-PC, G-FRAP, and G-DPPH represent the phenolic content and antioxidant activity of gastric supernatant; I-PC, I-FRAP, and I-DPPH represented the phenolic content and antioxidant activity of intestinal supernatant; G-SPC- soluble protein content in gastric supernatant; I-SPC- soluble protein content in intestinal supernatant; TDF- total dietary fibre of biscuits; SDF- soluble dietary fibre of biscuits; IDF- insoluble dietary fibre; UR-TPC, UR-FRAP, and UR-DPPH represented the total phenolic content and antioxidant activity of undigested residue; UR-β-glucan- β-glucan content of undigested residue.

Table S1. Formula of sorghum biscuits enriched with dried mushroom powders.

Biscuits	Sorghum flour (g)	Mushroom powder (g)	Sugar (g)	Vegetable shortening (g)	Salt (g)	Sodium bicarbonate (g)	Water (g)
CB	225	-	65	64	2.1	2.5	50
5% LEB	213.75	11.25	65	64	2.1	2.5	50
10% LEB	202.5	22.5	65	64	2.1	2.5	50
15% LEB	191.25	33.75	65	64	2.1	2.5	50
5% AAB	213.75	11.25	65	64	2.1	2.5	50
10% AAB	202.5	22.5	65	64	2.1	2.5	50
15% AAB	191.25	33.75	65	64	2.1	2.5	50
5% TFB	213.75	11.25	65	64	2.1	2.5	50
10% TFB	202.5	22.5	65	64	2.1	2.5	50
15% TFB	191.25	33.75	65	64	2.1	2.5	50

Table S2. Starch, protein and dietary fibre contents and *in vitro* glycaemic response of sorghum biscuits enriched with dried mushroom powders.

Biscuits	Starch (g/100g dw)	Protein (g/100g dw)	TDF (g/100g dw)	IDF (g/100g dw)	SDF (g/100g dw)	Glycaemic glucose (g/100g dw)
CB	43.39 ± 0.45[a]	7.21 ± 0.02[e]	8.23 ± 0.03[f]	6.70 ± 0.05[f]	1.53 ± 0.02[f]	39.94 ± 1.02[a]
5% LEB	42.08 ± 0.24[bc]	7.76 ± 0.02[c]	10.75 ± 0.02[e]	9.06 ± 0.05[e]	1.68 ± 0.04[f]	33.96 ± 0.92[b]
10% LEB	40.77 ± 0.21[de]	8.25 ± 0.11[b]	12.63 ± 0.32[d]	10.87 ± 0.29[d]	1.76 ± 0.03[f]	33.12 ± 0.22[b]
15% LEB	39.55 ± 0.31[f]	8.84 ± 0.05[a]	14.54 ± 0.51[c]	12.75 ± 0.49[bc]	1.79 ± 0.02[f]	32.27 ± 0.59[b]
5% AAB	41.81 ± 0.30[cd]	7.28 ± 0.02[de]	11.42 ± 0.20[e]	9.59 ± 0.30[e]	1.84 ± 0.10[ef]	32.82 ± 0.97[b]
10% AAB	38.91 ± 0.26[fg]	7.29 ± 0.05[de]	15.31 ± 0.12[bc]	12.90 ± 0.01[b]	2.42 ± 0.11[cd]	31.68 ± 1.56[b]
15% AAB	37.98 ± 0.43[g]	7.38 ± 0.07[d]	17.36 ± 0.34[a]	14.37 ± 0.32[a]	2.98 ± 0.03[ab]	27.35 ± 1.93[c]
5% TFB	42.99 ± 0.49[ab]	7.20 ± 0.01[e]	11.60 ± 0.21[de]	9.44 ± 0.25[e]	2.17 ± 0.04[de]	32.08 ± 1.19[b]
10% TFB	41.36 ± 0.47[cd]	7.31 ± 0.03[de]	14.48 ± 0.35[c]	11.81 ± 0.19[cd]	2.66 ± 0.16[bc]	30.80 ± 0.94[b]
15% TFB	39.94 ± 0.36[ef]	7.33 ± 0.05[de]	16.37 ± 0.16[ab]	13.13 ± 0.01[b]	3.24 ± 0.17[a]	26.63 ± 8.03[c]

Values in the same column with different letters are significantly different ($p < 0.05$). Values are means ± standard deviation (n=3). This table of supplementary data are shown in our submitted paper ''Utilisation of dried shiitake (*Lentinula edodes*), black ear (*Auricularia auricula*), and silver ear (*Tremella fuciformis*) mushrooms into sorghum biscuits manipulates the predictive *in vitro* glucose values in relation to variations in biscuit physical characteristics''. The method for the evaluation of the glycaemic glucose was according to the description of Wu, *et al.* [1].

1. Wu, G.; Hui, X.; Stipkovits, L.; Rachman, A.; Tu, J.; Brennan, M.A.; Brennan, C.S. Whey protein-blackcurrant concentrate particles obtained by spray-drying and freeze-drying for delivering structural and health benefits of cookies. *Innovative Food Science & Emerging Technologies* **2021**, *68*, doi:10.1016/j.ifset.2021.102606.
